# Nomogram to Predict Cognitive Dysfunction After a Minor Ischemic Stroke in Hospitalized-Population

**DOI:** 10.3389/fnagi.2021.637363

**Published:** 2021-04-14

**Authors:** Li Gong, Haichao Wang, Xiaofeng Zhu, Qiong Dong, Qiuyue Yu, Bingjie Mao, Longyan Meng, Yanxin Zhao, Xueyuan Liu

**Affiliations:** ^1^Department of Neurology, Shanghai Tenth People's Hospital, Tongji University, Shanghai, China; ^2^Department of Nursing, Huashan Hospial North, Fudan University, Shanghai, China; ^3^Nanjing Medical University, Nanjing, China

**Keywords:** trimethylamine-N-oxide, nomogram, minor stroke, post-stroke cognitive impairment, cognitive dysfunction

## Abstract

An easily scoring system to predict the risk of cognitive impairment after minor ischemic stroke has not been available. We aimed to develop and externally validate a nomogram for predicting the probability of post-stroke cognitive impairment (PSCI) among hospitalized population with minor stroke. Moreover, the association of Trimethylamine N-oxide (TMAO) with PSCI is also investigated. We prospectively conducted a developed cohort on collected data in stroke center from June 2017 to February 2018, as well as an external validation cohort between June 2018 and February 2019. The main outcome is cognitive impairment defined as <22 Montreal Cognition Assessment (MoCA) score points 6 – 12 months following a minor stroke onset. Based on multivariate logistic models, the nomogram model was generated. Plasma TMAO levels were assessed at admission using liquid chromatography tandem mass spectrometry. A total of 228 participants completed the follow-up data for generating the nomogram. After multivariate logistic regression, seven variables remained independent predictors of PSCI to compose the nomogram included age, female, Fazekas score, educational level, number of intracranial atherosclerotic stenosis (ICAS), HbA1c, and cortical infarction. The area under the receiver-operating characteristic (AUC-ROC) curve of model was 0.829, C index was good (0.810), and the AUC-ROC of the model applied in validation cohort was 0.812. Plasma TMAO levels were higher in patients with cognitive impairment than in them without cognitive dysfunction (median 4.56 vs. 3.22 μmol/L; *p* ≤ 0.001). In conclusion, this scoring system is the first nomogram developed and validated in a stroke center cohort for individualized prediction of cognitive impairment after minor stroke. Higher plasma TMAO level at admission suggests a potential marker of PSCI.

## Introduction

Post-stroke cognitive impairment (PSCI) causes a great burden to stroke survivors. Even minor stroke survivors are at increased risk of developing cognitive impairment (Gong et al., 2020), affecting executive function, speech ability. However, due to the absence of disabling conditions, they are more likely to be neglected for their cognitive dysfunction. Cognitive impairment at acute stage of non-disabling ischemic stroke has been related to advanced age, educational level, severity of intracranial atherosclerotic stenosis (ICAS), infarct location, and evidence of white matter hyperintensity (WMH). Our previous finding has suggested intestinal microbiota may play an important role in the cognitive performance post-stroke (Liu et al., 2020). Trimethylamine N-oxide (TMAO) is a metabolite generated primarily from dietary choline, phosphatidylcholine, and L-carnitine through the action of gut microbiota, and is a potential novel risk factor for stroke severity (Wu et al., 2020), but its relation to cognitive dysfunction after minor stroke has been less well-established.

Cognitive performance may vary from the acute to chronic stages of stroke, but few studies have used externally validated models to predict PSCI (Kandiah et al., 2016; Chander et al., 2017), and there is a need for risk scoring system to predict PSCI for individual minor stroke survivor. Nomogram is an useful tool for clinicians to make a visualized and quick risk assessment, and has been widely used for clinical decision-making in a particular patient. However, this model has only recently been applied to predict stroke outcomes (Busch et al., 2018), and is needed for validating in PSCI. The main objective of this study was to develop and externally validate a nomogram model to predict the potential risk of cognitive dysfunction beyond 6-month minor stroke.

## Methods

### Study Design, Participants, Patient Consents

This was a longitudinal, prediction model development and validation study, which included data in stroke center from June 2017 to February 2018. Participants were enrolled if they met the following criteria: (1) age 18 or older, (2) diagnosed with acute ischemic stroke based on diffusion-weighted magnetic resonance imaging (MRI) within 2 weeks, (3) National Institutes of Health Stroke Scale (NIHSS) score under 5, (4) no history of psychiatric disorders or diagnosis of cognitive impairment before the onset of the current stroke, and (5) complete data for all variables of interest. Validation cohort included subjects, admitted between June 2018 and February 2019, from the same stroke center. The same inclusion/exclusion criteria were applied to the external validation cohort. The study was approved by the Ethics Committee of Shanghai Tenth People's Hospital (Shanghai, China) and was conducted in accordance with the Declaration of Helsinki principles. All of the participants and their caregivers provided written informed consent.

### Outcome and Cognitive Assessment

The outcome measure was cognitive impairment defined as <22 Montreal Cognition Assessment (MoCA) score points 6–12 months following a minor stroke onset. Cognitive status was evaluated by an experienced neurologist via structured clinical interview and the MoCA, a sensitive and widely used measure of PSCI as our previous work described. The scores of the MoCA scale range from 0 to 30, with higher scores indicating a better cognitive function. Our goal was to develop a model that clinician could use to predict any cognition decline after minor stroke.

### Predictors and Sample Collection

The demographic and clinical data were collected at the admission as our previously published study (Gong et al., 2020). Several neuroimaging variables were also evaluated: intracranial stenosis as a narrowing exceeding 50% of the luminal diameter by MRA, severity white matter hyperintensity (WMH) by Fazekas scores, and the distribution of lesions (cortical, subcortical, deep area, and sub-tentorium).

Fasting serum samples were routinely performed on the second day after admission to the hospital for routine biochemical tests and TMAO levels from validation cohort. Whole-blood samples were centrifuged into plasma, separated into vials, and stored in a −80°C refrigerator until analysis. All chemicals and solvents were analytical or HPLC grade. Water, methanol, acetonitrile, formic acid was purchased from Thermo Fisher Scientific (Thermo Fisher Scientific, Waltham, MA, USA). Chloroform was from Sinopharm Chemical Reagent Co., Ltd. (Shanghai, China). Firstly, 100 μL of sample was added to a 1.5 mL Eppendorf tube, 300 μL of ice-cold mixture of methanol and acetonitrile (2/1, v/v) (containing 0.01 mol/L BHT) was added, and the mixtures were vortexed for 30 s, ultrasonicated at ambient temperature for 10 min, stored at −20°C for 30 min. The extract was centrifuged at 13000 rpm, 4°C for 15 min. 200 μL of supernatant in a glass vial was dried in a freeze concentration centrifugal dryer 200 μL mixture of methanol and water (2/98, v/v) were added to each sample, then samples vortexed for 30 s and ultrasonicated at ambient temperature for 2 min. 200 μL chloroform were added to each sample, stored at 4°C for 10 min. Samples were centrifuged at 13000 rpm, 4°C for 5 min. The supernatants (100μL) from each tube were collected using crystal syringes, filtered through 0.22 μm microfilters and transferred to LC vials. The vials were stored at −80°C until LC-MS analysis. An AB ExionLC (AB SCIEX, Framingham, MA) coupled with an AB SCIEX API 6500 Qtrap+ System (AB SCIEX, Framingham, MA) was used to analyze the metabolic profiling in ESI positive and negative ion modes and AB SCIEX OS workstation (version 1.7.1). A Waters UPLC HSS T3 column (1.8 μm, 2.1 ×100 mm) were employed in positive and negative ion modes. The binary gradient elution system consisted of (A) water (containing 0.1 % formic acid, v/v) and (B) acetonitrile and separation was achieved using the following gradient: 0 min, 0% B; 1 min, 0% B; 3.5 min, 100% B; 4.5 min, 100% B; 4.51 min, 0% B; 6 min, 0% B. All the samples were kept at 4°C during the analysis. The injection volume was 5 μL. Using an electrospray ion source (ESI), the analyte was analyzed in a multi-reaction detection (MRM) mode under positive and negative ion modes scanning, which greatly improved sensitivity. At the same time, the mass spectrometry parameters such as DP and CE were optimized, and the target compound ion pair can be quickly screened and determined under the optimal conditions. The optimized mass spectrum analysis conditions were as follows: positive mode: collision gas 35; ion spray voltage: 5500 V; ion spray temperature: 600°C; ion source gas1: 60; gas2: 50;The QCs were injected at regular intervals (every 6~8 samples) throughout the analytical run to provide a set of data from which repeatability can be assessed.

### Follow-Up

A total of 269 patients enrolled in the present study were scheduled for out-patient follow-up 6–12 months. The same neuropsychological tests were performed by an experienced neuropsychologist who was blinded to the medical records of the participants. Finally, 228 subjects (85%) took part in the follow-up visit. The remaining 41 subjects were lost in the follow-up list due to the following reasons: two death, 18 away from Shanghai, and 21 refusal. As for the validation cohort, a total of 66 participants completed the follow-up visit, with four subjects lost, two way from Shanghai and three refusal (Supplementary Figure 1).

### Statistical Analysis

The data are presented as mean ± standard deviation for continuous quantitative variables, and as frequencies and rate (%) for categorical variables. Initially, the Kolmogorov- Smirnov test was applied to detect normal distributions among the quantitative variables. Subsequently, we used either Student's *t*-test to compare the normally distributed quantitative variables, and Chi-square test for the normally distributed qualitative variables. Mann-Whitney U test was applied to compare variables with a non-normal distribution. Multivariate logistic regression analysis was performed to evaluate the strength of the aforementioned association according to the odds ratio (OR) and corresponding 95% confidence interval (CI), using the forward Wald method, with the F probability of entry set at 0.05 and that of removal set at 0.10. Variables with *p* <0.05 in multivariate analysis were incorporated into R language to establish the nomogram of the prediction model. To discriminate patients with and without PSCI, area under the receiver-operating characteristic curve (AUC-ROC) was accessed to calculate the predictive accuracy of the nomogram model. Then Bootstrap method (1,000 times of resampling) was used for internal verification to calculate the corrected C index, which is equivalent to AUC-ROC, ranging from 0.5 to 1.0, with higher score indicating better predictive accuracy. The model built from the development cohort was then applied to the validation cohort and performance was also assessed by AUC-ROC. Calibration of the risk prediction model was assessed in the development cohort by the plot comparing the observed probability of PSCI according to the total score of the nomogram against the predicted probability based on the nomogram and by using the Hosmer-Lemeshow test that assesses whether or not the observed event rates matched the expected rates in patients with minor stroke. Finally, the relationship between TMAO levels and PSCI in patients with minor stroke from validation cohort was evaluated by Mann-Whitney U test. A 2-tailed *P*-values <0.05 were considered statistically significant. R software, version 3.6.2 (2019 The R Foundation for Statistical Computing Platform) and SPSS 20(SPSS, Inc., USA), Prism 7 (2018 GraphPad Software, La Jolla, CA) were used for all data analysis.

## Results

### Characteristics of the Subjects in Development Cohort

Among 228 individual subjects who completed a follow-up visit (median follow-up time: 272 days), 122 subjects (53.5%) were identified as having PSCI. The average age of the patients was 62.61 ± 10.63 years old, including 162 males (71.1%), 149 patients with hypertension (65.4%), 73 patients with diabetes (32%), and 21 patients with hyperlipidemia (11%). The average length of education was 7.04 ± 4.94 years, and NIHSS score was 1.91 ± 1.18. Age, gender, education, hypertension, NIHSS score, Fazekas score, number of ICAS, cortical infarcts, HbA1c were found to be significantly different between PSCI (MoCA <22) and non-PSCI (MoCA≥22) groups (Table 1) in univariate analysis (*p* <0.05) and were included in the initial regression model.

**Table 1:** Characteristics and univariate comparison of PSCI (MoCA < 22) and non-PSCI (MoCA ≥ 22) groups in development cohort.

**Variables**	***n* = 228**	**MoCA < 22 (*n* = 122)**	**MoCA ≥ 22 (*n* = 106)**	***p*-value**
Age (mean ± SD, years)	62.16 ± 10.63	64.32 ± 9.82	60.55 ± 11.03	0.018*
Sex (male, %)	162 (71.1)	80 (65.6)	82 (77.4)	0.027*
Education (mean ± SD, years)	7.04 ± 4.94	10.38 ± 3.42	7.04 ± 4.94	0.033*
NIHSS score (mean ± SD)	1.91 ± 1.18	2.08 ± 1.18	1.72 ± 1.15	0.021*
mRS score of 0-2, n(%)	161 (70.6)	80 (65.6)	81 (76.4)	0.073
Intravenous thrombolysis, *n* (%)	49 (18.2)	21 (19.6)	28 (17.3)	0.626
**History of disease and medication**, ***n*** **(%)**				
TIA or prior stroke	58 (25.4)	28 (23.0)	30 (28.3)	0.355
Hypertension	149 (65.4)	89 (73.)	60 (56.6)	0.010*
Diabetes	73 (32.0)	43 (35.2)	30 (28.3)	0.262
Hyperglycaemia	25 (11.0)	18 (14.8)	7 (4.4)	0.049*
Atrial fibrillation	11 (4.8)	7 (35.7)	4 (3.8)	0.490
Use of antihypertensives	141 (61.8)	86 (70.5)	55 (51.9)	0.004*
Use of antithrombotics	21 (9.2)	12 (9.8)	9 (8.5)	0.726
Use of lipid-lowering drugs	24 (10.5)	17 (13.9)	7 (4.4)	0.072
Use of anti-diabetics	66 (28.9)	40 (32.8)	26 (24.5)	0.170
Current or previous smoking	135 (59.2)	70 (57.3)	65 (61.3)	0.672
Current or previous drinking	77 (33.8)	41 (33.6)	36 (40.0)	0.550
**Laboratory tests**				
TC, mmol/L	4.36 ± 1.08	4.34 ± 1.16	4.39 ± 0.97	0.672
TG, mmol/L	1.92 ± 1.37	1.90 ± 0.97	1.93 ± 1.28	0.242
LDL, mmol/L	2.24 ± 0.92	2.21 ± 0.97	2.28 ± 0.86	0.537
HDL, mmol/L	1.12 ± 0.71	1.01 ± 0.25	1.15 ± 1.00	0.390
FPG, mmol/L	6.35 ± 2.40	6.45 ± 2.44	6.23 ± 2.38	0.708
HbA1c, mg/dL	6.81 ± 1.70	7.88 ± 1.86	6.74 ± 1.48	0.037
Hcy, umol/L	11.15 ± 8.64	11.17 ± 10.36	11.19 ± 7.84	0.065
Uric acid, umol/L	326.08 ± 98.01	324.66 ± 102.90	327.71 ± 92.51	0.815
**Neuroimaging characteristics**				
Fazekas score (mean ± SD)	2.20 ± 1.69	2.23 ± 1.39	1.23 ± 2.38	0.028*
ICAS ≥ 50%, *n* (%)	100 (43.9)	60 (49.2)	40 (37.7)	0.011*
ICAS number	1.32 ± 0.84	2.23 ± 1.39	1.23 ± 2.38	0.003*
OCSP (ACI, %)	146 (64.0)	35 (53.0)	111 (48.7)	0.221
**Distribution of infarcts**				0.017*
Cortical	42 (18.4)	31 (25.4)	11 (10.4)	0.003*
Sub-cortical	64 (28.1)	34 (27.9)	30 (28.3)	0.942
Deep area	68 (29.8)	29 (23.8)	39 (36.8)	0.032
Subtentorial	54 (23.7)	28 (23.0)	26 (24.0)	0.780

PSCI, post-stroke cognitive impairment; TIA, transient ischemic stroke; NIHSS, National institute of Health Stroke Scale; mRS, modified ranking scale; ICAS, intracranial atherosclerosis stenosis; TC, total cholesterol; TG, total triglyceride; LDL, low density lipoprotein; HDL, high density lipoprotein; FPG, fasting plasma glucose; HbA1c, glycated hemoglobin; Hcy, homocystine; OCSP, Oxfordshire Community Stroke Project; ACI, anterior cerebral infarction. *p < 0.05.

### Development of an Individualized Prediction Model

**Figure 1:**
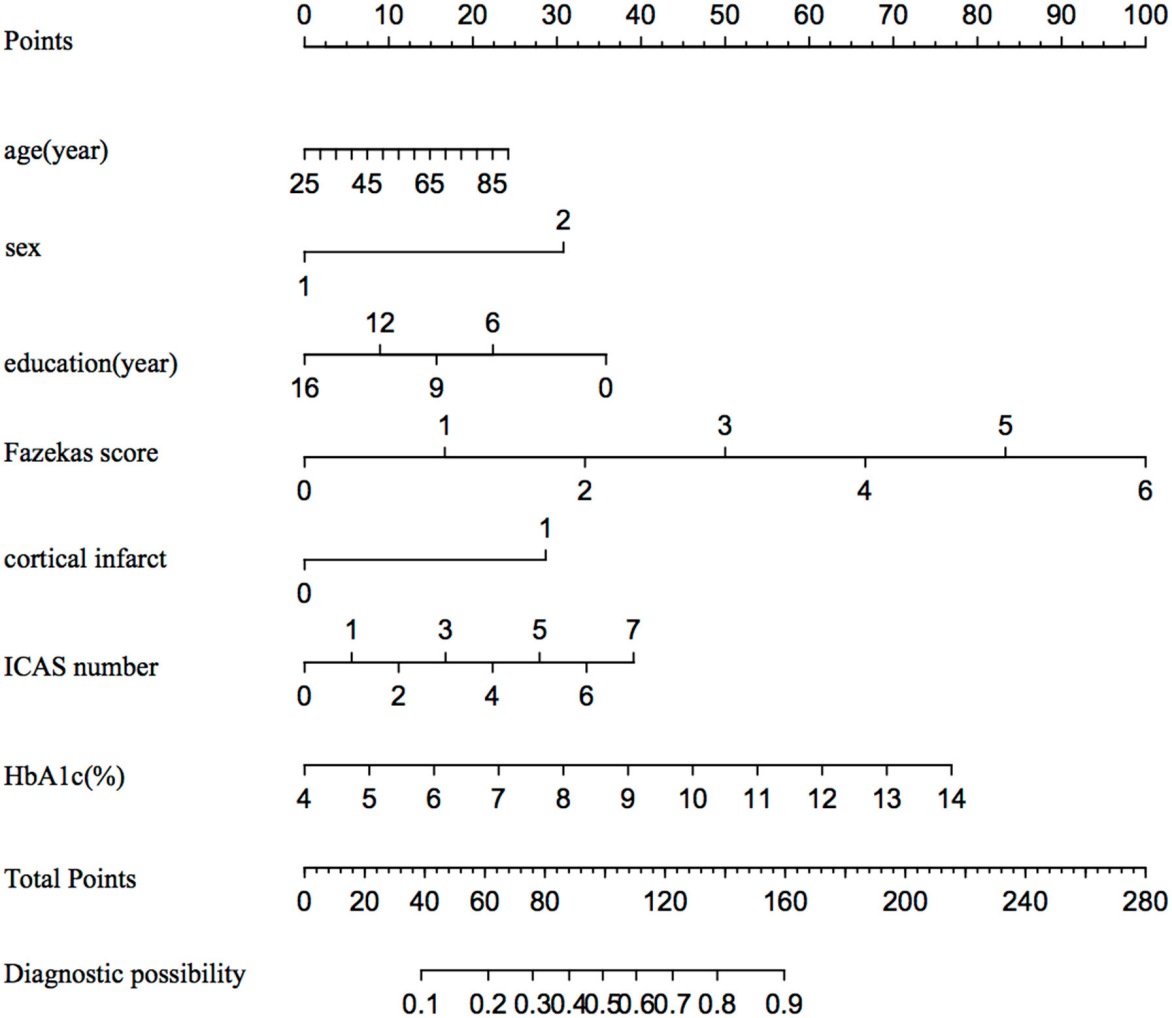
The nomogram for predicting the probability of PSCI among minor stroke. ICAS indicates intracranial atherosclerosis stenosis; and HbA1c, glycated hemoglobin.

**Figure 2:**
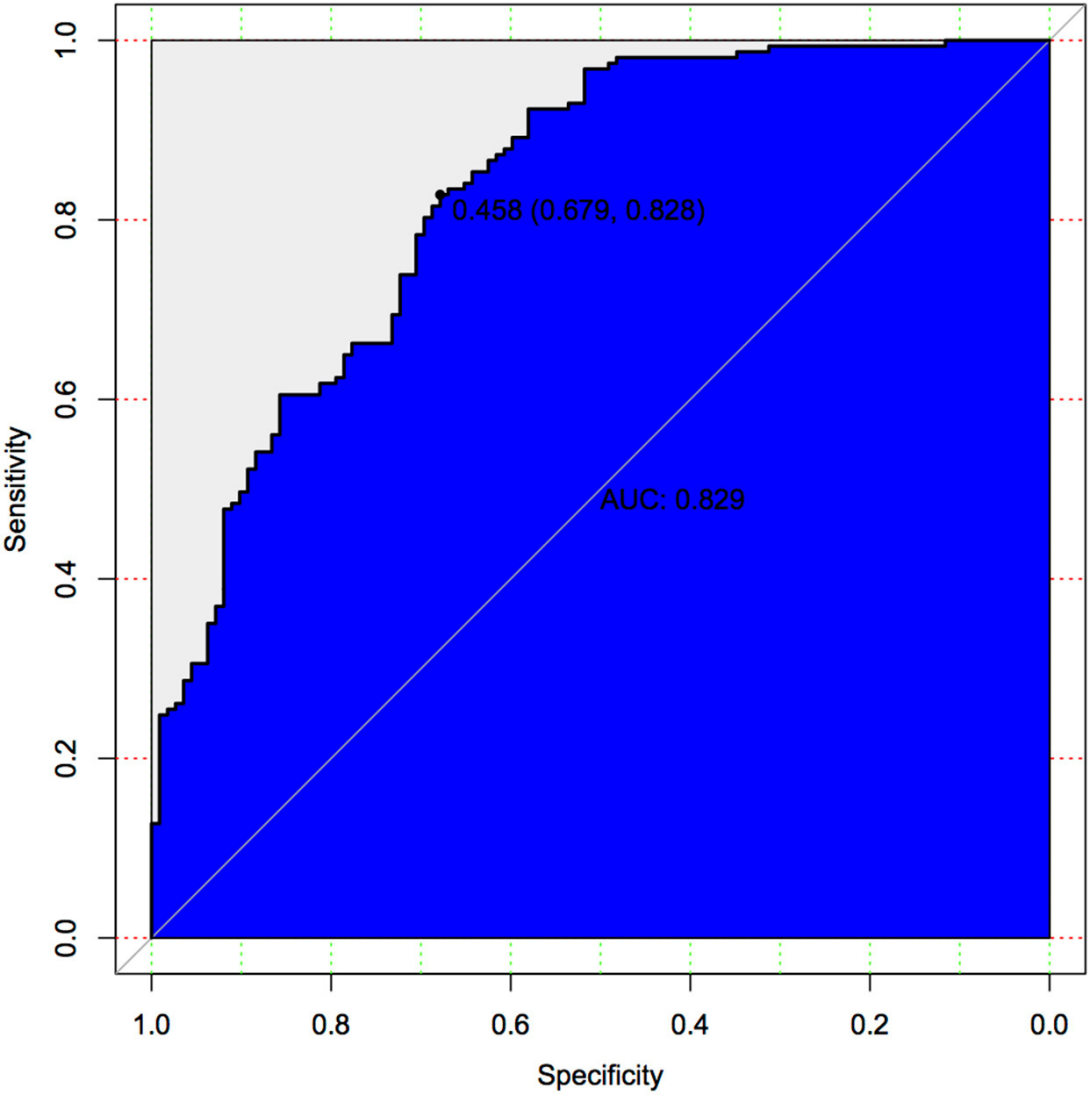
Predictive model based on logistic analysis for early diagnosis of PSCI in development cohort.

In binary logistic regression model, hypertension and NIHSS score were eliminated for their little significance. As shown in Table 2, seven potential predictors yielded by the binomial logistic regression model (LR method) were considered for model development: age (OR1.032, 95%CI 1.002–1.063), female (OR1.032, 95%CI 1.002–1.063), Fazekas score (OR 1.181, 95%CI 1.018–1.369), educational level (OR 0.937, 95%CI 0.883–0.993), number of ICAS (OR 1.070, 95%CI 1.070–1.733), HbA1c (OR 1.228, 95%CI 1.023–1.475), and cortical infarction (OR5.556, 95%CI 1.427–21.635). Furthermore, A prediction model was established using the nomogram on the basis of these seven factors. A summary of the point value of each factor used to calculate the total score presented in Figure 1. The area under the ROC curve of the development model was 0.829, with sensitivity 67.9%, specificity 82.8% (Figure 2).

**Table 2:** Descriptive statistics and adjusted association between each predictor and PSCI in development cohort.

					**95% CI of OR**	
	**β**	***S.E***	***p-*value**	**OR**	**Lower**	**Upper**
**Age**	0.031	0.015	0.035*	1.032	1.002	1.063
**Age(female)**	−0.710	0.281	0.011*	2.035	1.173	3.530
**Fazekas score**	0.166	0.075	0.028*	1.181	1.018	1.369
**Cortical infarcts**	1.715	0.694	0.013*	5.556	1.427	21.635
**Education**	−0.066	0.030	0.029*	0.937	0.883	0.993
**ICAS number**	0.309	0.123	0.012*	1.362	1.070	1.733
**HbA1c**	0.205	0.093	0.028*	1.228	1.023	1.475

PSCI, post-stroke cognitive impairment; ICAS, intracranial atherosclerosis stenosis; HbA1c, glycated hemoglobin. *p < 0.05.

### Apparent Performance of the PSCI-Risk Nomogram and External Validation

**Figure 3:**
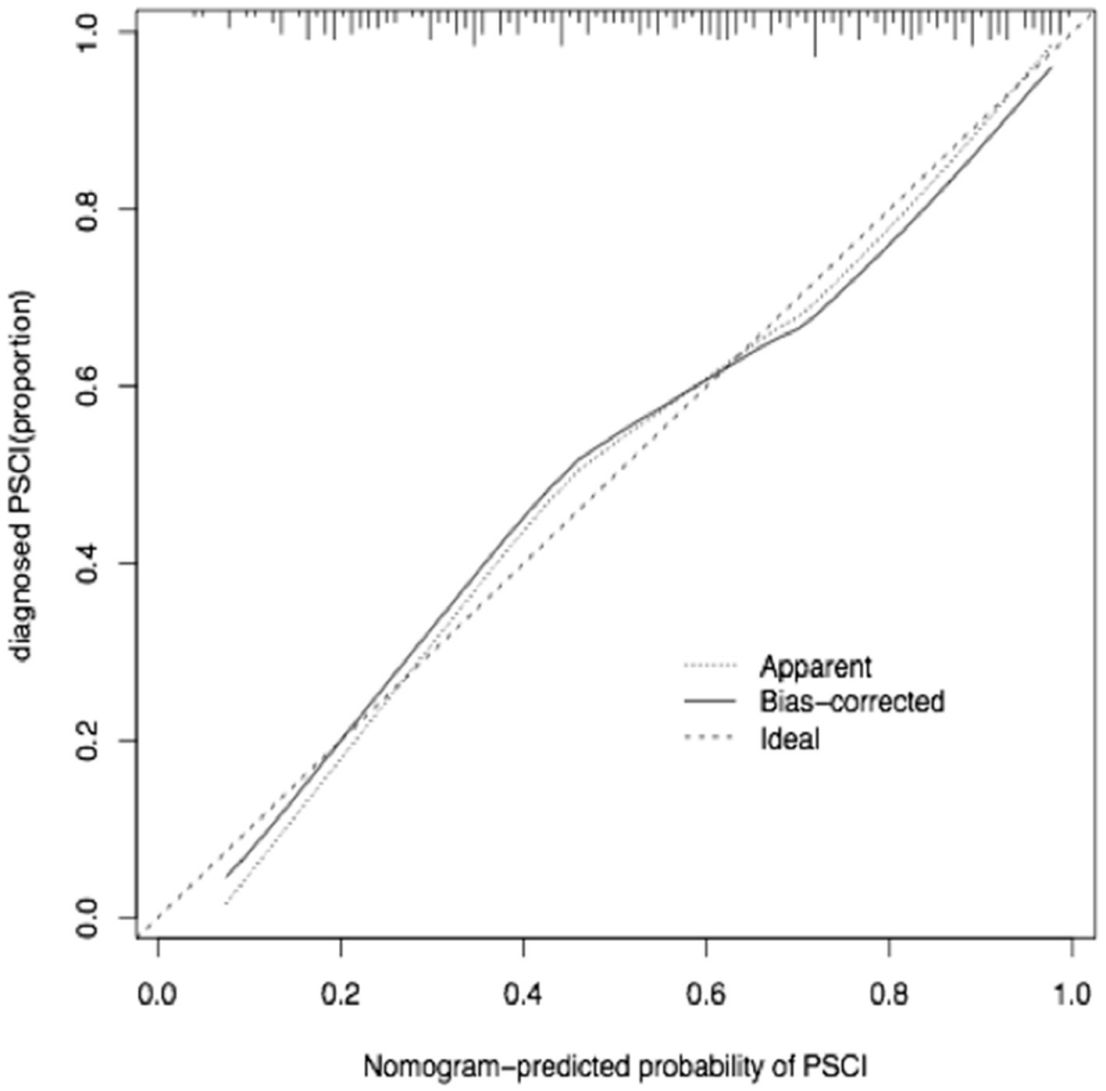
Calibration curve for nomogram-predicted probability of PSCI in minor-stroke patients.

**Figure 4:**
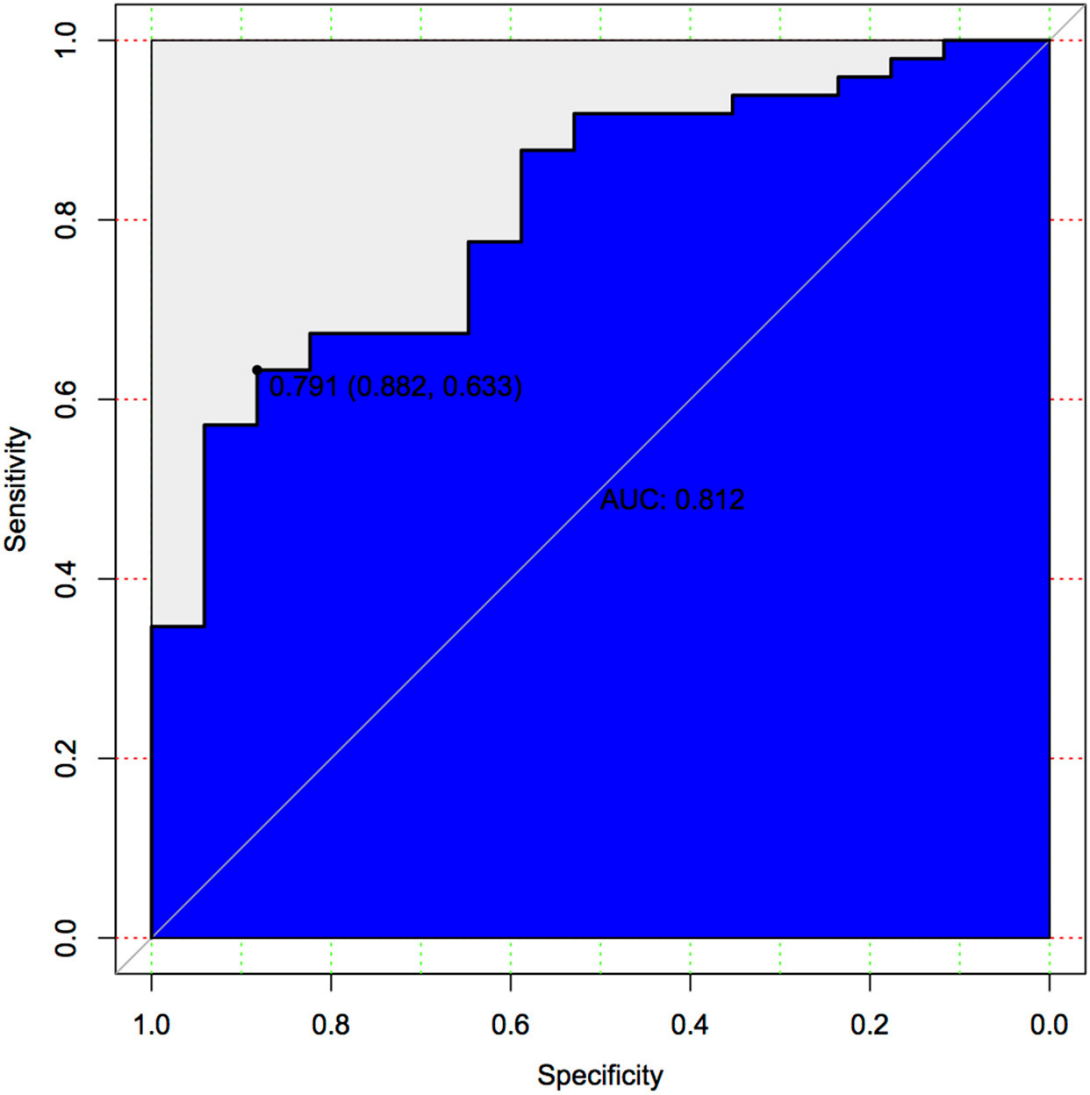
Predictive model based on logistic analysis for early diagnosis of PSCI in validation cohort.

The calibration curve of the nomogram for the predicted probability of PSCI in patients with minor stroke demonstrated good agreement in this cohort (Figure 3). The results of the 1000 bootstrap samples estimated the AUC to be 0.810, which suggested the model's good discrimination. As shown in Figure 4, the area under the ROC curve of the validation cohort was 0.812, which presents 88.2% of sensitivity, 63.3% of specificity.

### Relationship Between TMAO and Post-stroke Cognitive Impairment

**Figure 5:**
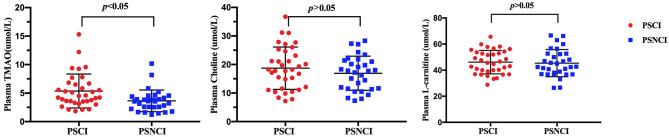
Comparison of serum TMAO, choline, and L-carnitine levels between minor-stroke patients with and without PSCI. PSCI indicates post-stroke cognitive impairment; and PSNCI, post-stroke non-cognitive impairment.

In validation cohort, blood samples of all 66 patients were collected, plasma TMAO levels were higher in patients with cognitive impairment than in patients without cognitive dysfunction after 6 months of a stroke onset (median 4.56 vs. 3.22 μ mol/L; *p* ≤ 0.001). However, there were no significant difference in L-carnitine and choline levels (Figure 5).

## Discussion

This study presented and externally validated the nomogram based upon the age stage, sex, years of education, WMH score, severity of intracranial atherosclerotic stenosis, infarct location, and HbA1c level to predict the probability of cognitive dysfunction following minor stroke. The model provides clinicians with practical tool for quick and individualized prediction of cognitive performance after minor stroke using readily available clinical information.

To date, the most widely accepted time point of PSCI evaluation has been under debate, which ranges from 3 to 6 month (Levine et al., 2015; Chander et al., 2017). The reason for choosing 6–12 months after stroke as the observation point is mainly based on two reasons. First, repeated evaluation of cognitive status by MoCA scale within relatively short time, such as 3 months, may be affected by the learning effect, which commonly results in false negative outcome. Second, our previous finding shows incidence of PSCI at acute phase has reached a high level (>50%) (Gong et al., 2020). Therefore, we chose the 6 to 12 months as time point to evaluate PSCI for consideration that the cognitive condition of survivors may be in a relatively stable status during this time.

Consistent with existing findings, demographic variables (age, sex, years of education) played important roles in predicting PSCI (Ojala-Oksala et al., 2012; Gong et al., 2020). In addition, imaging variables are corroborated by the existing literature identified as being important risk factors for PSCI. The strongest predictor of PSCI in our model is WMH as previously reported. The relationship between WMH and cognitive decline was similar to the CHANGE score (Kandiah et al., 2016; Chander et al., 2017), as well as similar to the association between cerebral small vascular disease burden and cognitive performance (Yang et al., 2015; Jiang et al., 2019). In contrast, this relationship between chronic brain lesion and cognitive dysfunction is not supported by the outcome of the study that enrolled patients with a minor stoke/TIA (Mandzia et al., 2016). This difference might be explained by the fact that 46 percent of 92 patients were diffusion-weighted imaging positive, and assessment of their specific domains of executive function, psychomotor processing speed, but not assessed by MoCA. It has been widely accepted that oxidative stress and neuroinflammation have pathological roles in cognitive dysfunction. Due to the decreased antioxidative nature, brain white matter is more vulnerable to the oxidative stress following a stroke onset, contributing to loss of white matter integrity and cognitive impairment (Besga et al., 2017; Boots et al., 2020). In addition, the present data revealed higher risk of cognitive dysfunction was not only associated with WMH score and cortical infarcts (Saczynski et al., 2009), but also with the number of ICAS. This positive association between ICAS and cognitive impairment not only supports, but also extends our previous cross-sectional finding. ICAS may suggest systemic microcirculation dysfunction, higher resistance in small vessels, and impaired vascular reactivity, finally resulting in decreased cerebral perfusion (Zhu et al., 2014; Gong et al., 2020). In fact, few studies have explored the association of HbA1c level with cognitive dysfunction after minor stroke, while a recent population-based cohort study has emphasized the contributions of diabetes (HbA1c ≥ 6.5%), but not prediabetic stage (HbA1c ≥ 5.7%), in post-stroke dementia (Shang et al., 2020). Although inclusion of other variables and more detailed characteristics (e.g., sample of cerebrospinal fluid, fMRI data, and PET-CT) may increase the discriminative ability of our model, we intentionally limited the set of predictors to demographic and imaging/blood variables readily available in most stroke centers. This could make the present model more practical, and thereby permit it more universal in the course of stroke management, which is the most valuable part of this model.

An important result in the present study was the establishment of a nomogram model to predict PSCI, which has not been reported previously among survivors with minor stroke. Moreover, this model showed excellent discrimination and calibration when applied to the external validation cohort. The calibration belt indicated that predicting power generated this model is as good as to represent actual risk. Although a few previous studies have examined the relationship between risk score and PSCI, there is growing evidence that nomograms have a better performance compared with risk scores (Cappellari et al., 2018, 2019). In contrast to risk group, a nomogram model provides a visualized and individualized estimate of the predicting probability of a specific outcome for an individual patient, as well as an important tool of medical decision making based on the individual's disease characteristics. Thus, this model showing good predicting ability suggests that it is suitable for clinicians to have readily evaluation of the probability of cognitive decline before discharge.

Although our primary goal was to develop a nomogram model to predict PSCI, we also investigated the relationship between TMAO levels and cognitive decline. Our results demonstrated an association between higher plasma TMAO levels at admission and increased risk of PSCI. The present findings are partly in line with a recently 1-year longitudinal study that included 256 patients with acute ischemic stroke and reported an association of increased plasma TMAO levels with PSCI (Zhu et al., 2020). However, it should be noted that no measures of dietary intake or biochemical precursors of TMAO are available in numerous prior studies (Yin et al., 2015; Olek et al., 2019; Wu et al., 2020), which can directly affect TMAO levels. For that, in the present study, we measured these important precursors such as L-carnitine and choline, and phosphatidylcholine, but did not find an association of PSCI with them. Thus, it further suggests that serum concentration TMAO may be a metabolic marker of intestinal microbiota independently related with cognitive dysfunction beyond a minor stroke. Although previous studies have reported that TMAO may involve different processes of stroke pathogenesis including cholesterol metabolism, platelet reactivity, and glucose tolerance, we consider diversity of gut microbiota is more likely to explain the mechanism of our results partly because of our prior published finding (Liu et al., 2020). Furthermore, a recently novel research indicated that the alteration of gut microbiota composition contributes to pro-inflammatory microglia activation, leading to dementia-associated neuroinflammation (Wang et al., 2019), which may be another evidence explaining the present result. However, the relationship should be interpreted cautiously for the small sample size, and needs to be confirmedzin the future with a larger sample size.

The major strengths of our study include the longitudinal hospital-based design with a relatively low ratio of loss-up, as well as the good predicting ability validated by an external cohort. In addition, we use the same 3T MRI scanner for its sufficient sensitivity to detect either small vessel disease such as WMC, small infarcts, or ICAS. These lesions have great influence on cognitive impairment. However, this study has some limitations to consider. Firstly, the relationship between PSCI and plasma levels of TMAO may be dynamic and influenced by multiple variables such as diet, gut microbiota, but we cannot exclude the influence of these potential confounders due to the small number of external validation cohort. The serum TMAO level in this study was detected at the day after admission to stroke center in the validation cohort and it must be better to conduct dynamic comparison of the results after 6 months, which is warranted in our further research. Secondly, even though we have tried to exclude the prior cognitive impairment in any kinds, the concept of PSCI overlaps with AD, even some other neurodegeneration diseases like FTD. In that way, it should be noted it is difficult to exactly differentiate whether vascular cognitive impairment or early AD causes PSCI in this cross-sectional study, for that they commonly share similar risk factors, such as existing vascular damage and diabetes. Indeed, inclusion of more variables such as Apolipoprotein E (ApoE) or PET-CT may increase the predicting power. However, it should be noted that this could limit the practice and availability of the present model because those more detailed variables are not routinely tested in most stroke centers. What we are most concerned about in this study is to provide clinicians with a universal and quick tool, promoting early identification and timely intervention in the course of stroke management.

### Conclusion

This study provides an easy-to-use nomogram model to predict cognitive outcome beyond an minor stroke onset. This externally well-validated nomogram provides clinician a new tool more useful than traditional risk scores in patient counseling, because it can be readily and quickly applicable in presenting a visualized risk for each individual stroke survivor with an APP in mobile phone. In addition, we find the association of plasma TMAO level with PSCI, suggesting this metabolic marker linking intestinal microbiota to cognitive dysfunction. Future studies are warranted to investigate this potential target in cognitive impairment after minor stroke.

## Data Availability Statement

The original contributions generated for the study are included in the article/Supplementary Material, further inquiries can be directed to the corresponding authors.

## Ethics Statement

The studies involving human participants were reviewed and approved by the Ethics Committee of Shanghai Tenth People's Hospital (Shanghai, China) and was conducted in accordance with the Declaration of Helsinki principles. The patients/participants provided their written informed consent to participate in this study.

## Author Contributions

LG performed most of the experiments, interpreted data, and wrote the first draft of the paper. HW, XZ, and QD performed a part of experiments, analyzed the data, and collect blood samples. QD, QY, BM, and LM all took part to conceive the study and a part of sample collection. YZ critically edited the manuscript and supervised the study. XL mainly provided funding and designed the study. All authors contributed to the article and approved the submitted version.

## Conflict of Interest

The authors declare that the research was conducted in the absence of any commercial or financial relationships that could be construed as a potential conflict of interest.
