# Supplementary material for: Nomogram to Predict Cognitive Dysfunction After a Minor Ischemic Stroke in Hospitalized-Population
Source: Front Aging Neurosci. 2021 Apr 14;13:637363. doi: 10.3389/fnagi.2021.637363 (PMC8098660; doi:10.3389/fnagi.2021.637363)

Supplementary Table 1. Characteristics and comparison of development and validation cohorts

**Variables**	**development cohort (*n*=228)**	**validation cohort (*n*=66)**	***p* value**
Age (mean±SD, years)	62.61±10.63	64.92±9.33	0.090
Sex (male, %)	162(71.1)	53(80.3)	0.135
Education (mean±SD, years)	7.04±4.94	10.38±3.42	≤0.001*
NIHSS score (mean±SD）	1.91±1.18	3.02±2.31	0.539
Intravenous thrombolysis, *n* (%)	62.61±10.63	64.92±9.33	0.090
**History of disease and medication, *n* (%)**			
TIA or prior stroke	58(25.4)	15(22.7)	0.653
Hypertension	149(65.4)	46(69.7)	0.511
Diabetes	73(32.0)	22(33.3)	0.840
Hyperglycaemia	25(11.0)	9(13.6)	0.550
Atrial fibrillation	11(4.8)	2(3)	0.532
Current or previous smoking	135(59.2)	28(42.4)	0.016*
Current or previous drinking	77(33.8)	17(25.8)	0.219
**Laboratory tests**			
TC, mmol/L	4.36±1.08	4.38±0.99	0.988
TG, mmol/L	1.92±1.37	1.73±1.00	0.151
LDL, mmol/L	2.24±0.92	2.80±0.88	≤0.001*
HDL, mmol/L	1.12±0.71	1.06±0.22	0.091
FPG, mmol/L	6.35±2.40	6.85±2.88	0.318
HbA1c, mg/dL	6.81±1.70	7.18±1.92	0.173
**Neuroimaging characteristics**			
Fazekas score (mean±SD)	2.20±1.69	2.80±1.88	0.250
ICAS≥50%, *n* (%)	100(43.9)	32(48.5)	0.506
ICAS number	0.86±1.32	1.06±1.61	0.357
OCSP (ACI, %)	111(48.7)	35(53.0)	0.534
**Distribution of infarcts**			**0.476**
Cortical	42(18.4)	15(22.7)	0.437

Sub-cortical	64(28.1)	14(21.2)	0.266
Deep area	68(29.8)	15(22.7)	0.259
Subtentorial	54(23.7)	18(27.3)	0.550

TIA: transient ischemic stroke; NIHSS: National institute of Health Stroke Scale; ICAS: intracranial atherosclerosis stenosis; TC: total cholesterol; TG: total triglyceride; LDL: low density lipoprotein; HDL: high density lipoprotein; FPG: fasting plasma glucose; HbA1c: glycated hemoglobin; Hcy: homocystine; OCSP: Oxfordshire Community Stroke Project; ACI: anterior cerebral infarction. *$p<0.05$.

Figure 1. Flow chart of patients in development cohort and validation cohort. NIHSS: National institute of Health Stroke Scale

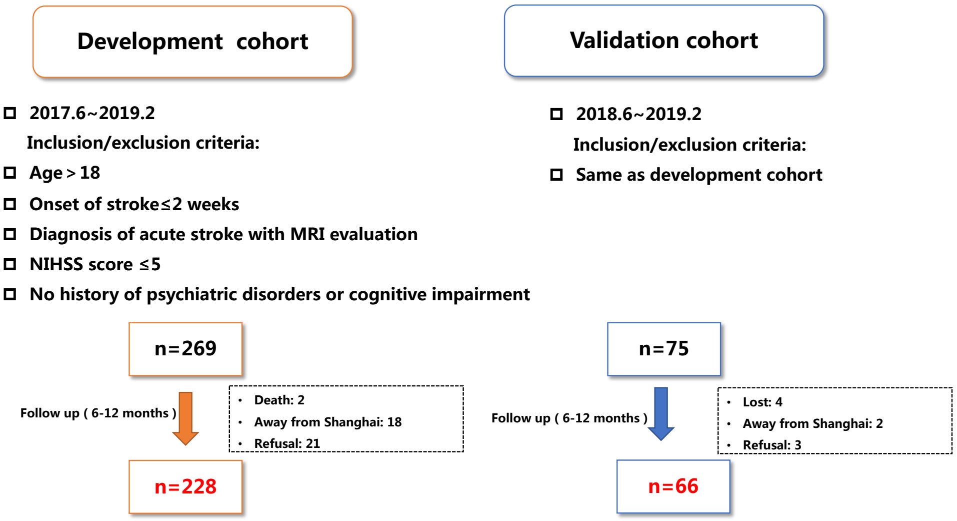

Supplement: Supplementary file 1 [file Data_Sheet_1.pdf]
